# Exploring the Influencing Factors for Contraceptive Use among Women: A Meta-Analysis of Demographic and Health Survey Data from 18 Developing Countries

**DOI:** 10.1155/2022/6942438

**Published:** 2022-11-14

**Authors:** Md. Akhtarul Islam, Md. Nafiul Alam Khan, Hasin Raihan, Sutapa Dey Barna

**Affiliations:** Statistics Discipline, Science Engineering and Technology School, Khulna University, Khulna, Bangladesh

## Abstract

**Background:**

The primary objective of this research was to investigate how socioeconomic and demographic factors influence the usage of contraceptives by women in 18 developing countries.

**Methods:**

The study used the latest DHS data from 18 developing countries in order to acquire a broad perspective of contraceptive methods. We applied meta-analysis techniques for 18 developing countries to find out the summary results.

**Results:**

The overall summary effect showed that the variable respondent education (OR = 1.39; 95% CI: 1.17 to 1.65), husband education (OR = 1.60; 95% CI: 1.32 to 1.93), type of place of residence (OR = 0.88; 95% CI 0.78 to 0.98), current working status (OR = 1.47; 95% CI 1.30 to 1.66), age of the respondent (OR = 3.41; 95% CI 2.35 to 4.93), breastfeeding status (OR = 1.34; 95% CI 1.11 to 1.62), and desire for more children (OR = 0.53; 95% CI 0.43 to 0.65) were the significant factors for contraceptive utilization in developing countries.

**Conclusions:**

According to the findings of this descriptive study, the respondent's age, level of education, and work status were shown to be the most significant factors that influence the usage of contraceptives in developing countries. It is necessary to take reasonable steps in order to increase the rate of utilizing methods of contraception among women of reproductive age who are uneducated, living in rural areas, and unemployed.

## 1. Introduction

It is projected that the world's population will reach 8.5 billion by the year 2030, 9.7 billion by the year 2050, and 11.2 billion by the year 2100. Most of the world's population growth will occur in sub-Saharan Africa and Asia, the regions of the globe with the lowest levels of development [1]. Therefore, rapid population growth might cause a diverse effect on the future economic growth of a country as well as cause constraints on the welfare of its citizens. Therefore, potential strategies need to be followed up to reduce this excessive growth and fertility rate. Using contraceptives is one of the effective methods of reducing fertility [2]. Contraceptives prevent women's conception through medical devices, drugs, or surgical procedures, which can reduce population growth and pregnancy-related morbidity and mortality [3–5]. Proper contraception can prevent unintended and unwanted pregnancy, which can reduce the risk of unintended child complicacy and unsafe abortions [6, 7]. About 44% of total annual pregnancy (approximately 227 million) is unplanned, and 56% of these unplanned pregnancies are terminated by abortion [8, 9]. As a result, nearly 56 million abortions are performed worldwide, with 50 million in developing countries [8]. These could be prevented if family planning and contraceptives were available and implemented in developing countries [10]. In developing countries, contraceptives can reduce unintended pregnancies and maternal mortality by 40% and prevent approximately 2.7 million infant deaths [11–13]. In 2015, around 64% of reproductive women used some type of contraception. Contraceptive use was significantly lower in the least developed countries (40%) and only 33% in Africa. Unmet contraceptive needs affect 24% of women in sub-Saharan African countries [14]. According to the Afghanistan Demographic and Health Survey (DHS), the primary reason for the rising fertility rate (5.2%/women) was the small range of contraceptive use [10]. Although there is no cost associated with obtaining modern methods of contraception in Albania, just 11% of Albanian women report ever having used any form of contraception [15]. However, according to the results of the Philippines National Demographic and Health Survey conducted in 2013, the country of the Philippines has a contraceptive prevalence rate of 55% (NDHS) [16]. According to a cross-sectional survey conducted in Egypt during 2017-2018, the percentage of married women currently using any kind of contraception was 38.3% [17].

Moreover, in 2017, approximately 214 million women in developing countries were interested in preventing pregnancy but were not using contraception [14]. In the case of Bangladesh, the use of contraceptives has increased drastically. “Task sharing is envisioned to create a more rational distribution of tasks and responsibilities among cadres of health workers to improve access and cost-effectiveness,” according to a WHO strategy for providing reproductive health services in low-income countries [18]. The Bangladesh Demographic and Health Survey (BDHS) that was conducted between 1993 and 1994 indicated that the contraceptive prevalence rate (CPR) had increased to 45% while the total fertility rate (TFR) had decreased to 3.4. [19]. The later report showed an increase in contraceptive use from 8% in 1975 to 62% in 2014 and a fall in TFR to 2.3 [20]. Thus, a significant decline has been seen in TFR during 1975-2014; still, women need to emphasize the use of contraceptives to reach our targeted CPR level of over 70% [5].

Sustainable Development Goal 3 (SDG) is aimed at ensuring universal access to sexual and reproductive health care services for healthy lives and well-being for all ages by the year 2030 [1, 21]. To fulfil this aim, this study was focused on finding the triggering factors influencing the utilization of contraceptives in developing countries. Previous studies have only concentrated on the factors related to contraceptives for a particular region. A comparative study on the factors behind contraceptive use in different countries using meta-analysis has not been investigated yet. Therefore, we applied meta-analysis techniques to the cross-sectional demographic and health survey data of 18 developing countries, including Bangladesh, to explore the summary results of the study on the influencing factors behind contraceptive use among women.

## 2. Materials and Methods

### 2.1. Design

We utilized meta-analysis approaches to the demographic and health survey cross-sectional data of 18 developing countries, including Bangladesh. Using meta-analysis, we then compared the outcomes between Bangladesh and 17 other developing countries.

### 2.2. Data Management

In the initial stage of this cross-sectional analysis, we utilized binary logistic regression to extract relevant data from a secondary dataset representative of the entire country, namely, the Bangladesh Demographic and Health Survey 2014 [20, 22]. We conducted a meta-analysis by making use of datasets from the Monitoring and Evaluating to Assess and Use Results from the Demographic and Health Survey (MEASURE DHS), which were only recently made public (in January 2020) [23, 24]. In addition, the most recent DHS data from 18 other developing countries, including Afghanistan, Albania, Bangladesh, Cambodia, Egypt, Ghana, Haiti, India, Indonesia, Jordan, Kenya, Maldives, Nepal, Pakistan, the Philippines, Senegal, Tanzania, and Timor-Leste, were adopted. The information in the DHS database originated from 91 different countries. We selected Bangladesh and 17 other developing countries since they were all similar in structure and had a comparable probability of sampling for data collection [25].

**Figure 1:**
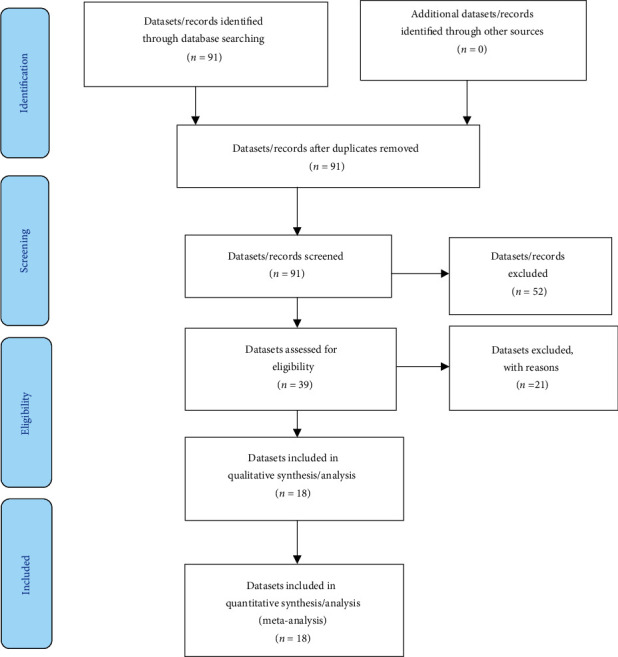
PRISMA (Preferred Reporting Items for Systematic Reviews and Meta-Analysis) flow diagram for the eligibility criteria of the datasets. We get a clear illustration of identifying and including DHS datasets for the random effects meta-analysis.

Figure 1 is a flowchart of the PRISMA method for choosing and using DHS datasets in random effects meta-analyses.

### 2.3. Variables

In this study, contraceptive utilization served as the dependent variable. The two unique levels were “yes” if the respondent used any contraceptive method and “no” if the respondent did not use any contraceptive method. We employed a collection of pertinent socioeconomic and demographic parameters as an independent variable to conduct the research and identify the affecting factors that were believed to cause infant and child mortality based on prior research. In contrast, the kind of a person's residence, whether or not they are breastfeeding, whether or not the mother was once employed, and whether or not they utilize contraceptives remained consistent with the existing category of DHS datasets.

The remaining covariates were further classified. For the meta-analysis, these variables were classed as educated or uneducated for study. Furthermore, for the meta-analysis with two categories, we recoded the variable (wealth status) as poor and wealthy. We combined the poorer and poorest categories and classified them as “yes” to indicate that the population was impoverished. In addition, we merged the categories “middle,” “richer,” and “richest” with “no,” which signifies individuals who live above the poverty line. For the meta-analysis, media access was classified as “yes” or “no.” The respondents' age was divided into two categories: 15–19 and 19–49.

### 2.4. Statistical Analysis

The study was carried out using the statistical software SPSS V.26 (SPSS Inc., Chicago, USA) and R V.3.6.2 (Bell Laboratories, New Jersey, USA). For the DHS data from 18 developing nations, we used meta-analysis [26]. Heterogeneity was assessed by enumerating values from *I*^2^ and *p* values among datasets [27]. We performed a random effects model in the meta-analytic approach as significant heterogeneity was found by which we estimated DerSimonian and Laird's pooled effect [28]. We also performed sensitivity analysis by omitting one country at a time. Supplementary File 1 shows the overall effect estimate changes after removing one study. Again, the random effects model was performed in the meta-analytical approach, and the forest plots were used to display the 95% CI, summary measure, and weight of each study for the most significant determinants. As a summary statistic, we utilized the odds ratio (OR), and all data was weighted to account for bias due to undersampling and oversampling. This study employed a “leave-one-study-out” sensitivity analysis [29, 30] to determine the strength of the results and to determine whether one country had a disproportionate effect on the meta-analysis.

## 3. Results

Table 1 displays the baseline characteristics of the selected factors for 18 developing countries.

An estimate of the average treatment effect that varies from study to study can be acquired by the true treatment effect from the random effects model, as has been illustrated in Tables 2 and 3. In this study, we intended to use the random effects model as the study showed high between-study variation (heterogeneity). About 97.2% of the variation (*I*^2^ = 97.2%) was observed for the type of place of residence. The overall OR was 0.88 (95% CI: 0.78 to 0.98), which means the individuals residing in rural areas were 12.5% less likely to use contraceptive methods than their urban counterparts. About 95.2% of the variation (*I*^2^ = 95.2%) was observed for respondent education. The overall OR was 1.39 (95% CI: 1.17 to 1.65), meaning the individuals with education were 39% more likely to use contraceptive methods than the respondents without education. Again, about 95.5% of the variation (*I*^2^ = 95.5%) was observed for the respondent's husband's education. The overall OR was 1.60 (95% CI: 1.32 to 1.93), which means the individuals with educated husbands were 60% more likely to use contraceptive methods than the respondents with uneducated husbands. Moreover, about 98.6% of the variation (*I*^2^ = 98.6%) has been observed for breastfeeding. The overall OR was 1.34 (95% CI: 1.11 to 1.62); i.e., the individuals who were breastfeeding their children were 34.3% more likely to use contraceptive methods than the respondents who were not breastfeeding their children. For the respondent's current working status, about 97.4% of the variation (*I*^2^ = 97.4%) was observed.

Here, the overall variation (*I*^2^ = 98.6%) of media exposure was about 98.6%, and the overall OR was 1.48 (95% CI: 1.25 to 1.76), which means the individuals with media access were 64.8% more likely to use contraceptive methods than the respondents with no media exposure. On account of the age of the respondents, about 98.4% of the variation (*I*^2^ = 98.4%) was observed. Hence, the overall OR was 3.41 (95% CI: 2.35 to 4.93), meaning the individuals aged more than or equal to 20 to 49 were 240.6% more likely to use contraceptive methods than the respondents less or equal.

About 96.6% of the variation (*I*^2^ = 96.6%) was observed, and the overall OR was 1.21 (95% CI: 1.10 to 1.35) for the respondent's wealth index, which infers that the individuals from the rich family background were 26.3% more likely to use contraceptive methods than the respondents from the low-income family background. Again, about 99.1% of the variation (*I*^2^ = 99.1%) was found for the desire for more children. The overall OR was 0.53 (95% CI: 0.43 to 0.65), concluding that the respondents who desired more children were 46.9% less likely to use contraceptive methods than the respondents who did not want more children.

**Figure 2:**
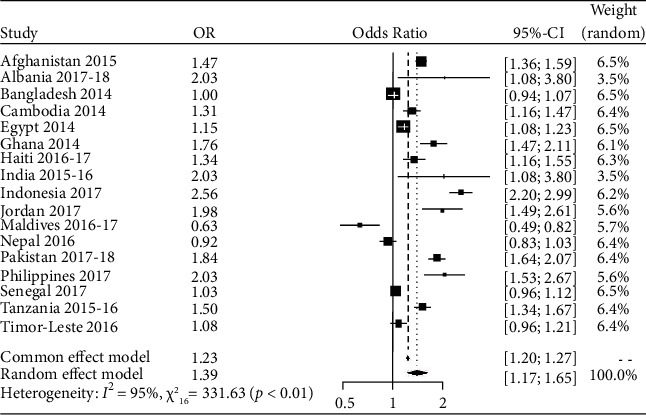
Forest plot for the variable respondent education.

In Figure 2, the shape of each box represents the study's weight, while each crossed line represents the 95% CI. The overall estimate of the random effects model for respondent education was 1.54; the 95% confidence interval for the overall estimate (1.25; 1.89) did not overlap with 1; hence, it can be inferred that educated women were 54% more likely to utilize contraceptive methods than uneducated women.

**Figure 3:**
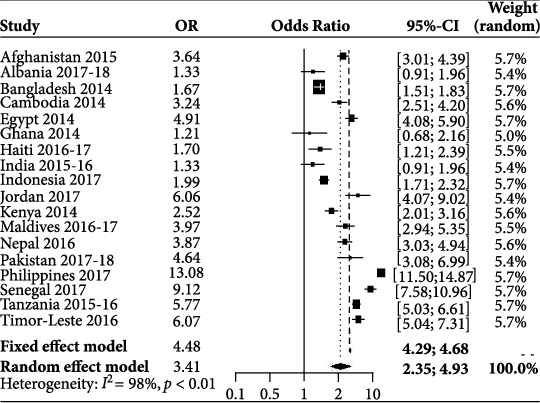
Forest plot for the variable respondent age.

In Figure 3, the shape of each box represents the significance of the study, and the lines that are crossed represent the 95% confidence intervals.

The overall estimate of the random effects model for respondent age was 3.41, and the 95% confidence interval of the overall estimate (2.35; 4.93) also did not overlap with one, which means that it was possible to draw the conclusion that women aged more than or equal to 20 years were 241 percent more likely to use contraceptive methods than women aged less than or equal to 19 years (see Table 4).

## 4. Discussion

The key findings of the meta-analysis showed that women's age, women's education, women's place of residence, current breastfeeding status, media access and wealth index, husband or partner's education, and women's working status were indeed the significant factors for contraceptive use among women. Additionally, the meta-analysis found that women's employment status was also a significant factor. Older women had more education, and those who lived in urban areas were more likely to be aware of the benefits of using the contraceptive method. Also, as media access and the education level of the husband or partner improved, so did their propensity to choose contraceptives. However, the findings reveal that those with lower incomes had a heightened awareness of the use of contraceptives. Both the ORs for Bangladesh in the meta-analysis model for women's education level and the overall OR for the meta-analysis were positive, indicating a similar positive relationship between women's education and the use of contraceptive methods. The OR for Bangladesh was 1.225, and the overall OR for the meta-analysis was 1.535. Women with lower or no levels of education were at a greater risk than women with higher levels of education. Women who finished school were more likely to have more than one job and to want to limit the number of children they had [31–33]. According to the findings of a meta-analysis that included 18 different nations, women of the elderly were more likely to use some form of birth control. When the age of the respondents was taken into account, prior research came to the same conclusions [8, 34, 35]. Our findings, however, did not coincide with those of a number of other studies, which found that age was not substantially related to the use of contraceptives [36–38]. In addition, people who came from wealthy homes were more likely to report using birth control. It was found that the wealth indexes with the least amount of money had the highest proportion of children who were unwanted. It was possible that different social and cultural ideas and the high cost of birth control were to blame for the low rate of low-income families using birth control [8, 39–41]. Women living in cities were more likely to use birth control than women living in rural areas. This could be because urban women had more accessible access to birth control options and were more likely to use them than women in rural areas [8, 42, 43]. The odds ratio from the meta-analysis showed that there was a favourable link between access to the media and the use of contraceptive techniques across all 18 nations. This was because women who had access to the media were more likely to know about the different ways to avoid getting pregnant [43, 44]. Those who wanted a more prominent family were much less likely to use contraception than those who were happy with their current family size [45]. According to the findings of this study, there was a correlation between women's employment and their use of birth control methods. A meta-analysis demonstrated that there was a positive connection between them. In earlier research, it was shown that there was a link between being employed and using birth control methods [33, 46, 47].

This study has both drawbacks and strengths. The DHS data in this analysis covered a broader range and time period, which is added to the selection bias. A variable was separated into two groups, and a cross-tabulation table was utilized to calculate the OR. Due to the absence of values or the insignificance of several independent variables in any of these countries, we were unable to account for some potential risk factors. Despite these limitations, the strength of our research was that we combined national survey cross-sectional data with meta-analysis data. This study explores the impact of different factors on the utilization of contraceptive methods across 18 countries, showing the variation of the effect over countries. Through integration, new information and insights have been developed, which will help the policymaker to make decisions quickly.

### 4.1. Implications for Policy and/or Practice

The study is aimed at exploring different factors influencing the utilization of contraceptives and their consistency among women within 18 developing countries, including Bangladesh using DHS data. Using meta-analysis techniques, this study focused on finding the actual and summary effects of different factors across developing countries so that the policymaker can focus the study's finding on reducing the risk of unintended child complicacy, unsafe abortions, and pregnancy-related morbidity and mortality.

## 5. Conclusion

The findings of this study revealed that significant factors for contraceptive use among women in developing countries include the respondent's age, maternal education, place of residence, media access and wealth index, husband or partner's education, and women's working status and desire for more children. Education for women was undoubtedly essential in raising people's consciousness about the importance of using birth control. This research thoroughly accepted the superiority of the rise in the average age of the respondents. Low-income families needed to receive extra attention for utilizing contraceptive methods. A particular emphasis needs to be placed on the disadvantaged rural women who lacked access to the media, was uneducated, and did not have jobs. To meet the Sustainable Development Goals and ensure that all women have access to sexual and reproductive health care by 2030, we need to implement programs that specifically help these women.

## Figures and Tables

**Table 1:** Baseline characteristic table for different selected variables for 18 developing countries.

Country name	Type of place of residence (*n*, %)		Respondent education level (*n*, %)		Currently breastfeeding (*n*, %)		Husband education level (*n*, %)		Respondent currently working (*n*, %)		Media exposure (*n*, %)		Respondent age (*n*, %)		Wealth index (*n*, %)		Desire for more children (*n*, %)		Contraceptive use (*n*, %)	
	Urban	Rural	No education	Has education	No	Yes	No education	Has education	No	Yes	No	Yes	15-19	20-49	Poor	Rich	Do not want	Want	Not using	Using
Afghanistan, 2015	6939 (23.9)	22131 (76.1)	16562 (57.0)	12508 (43.0)	29507 (89.1)	3163 (10.9)	16562 (57.0)	12508 (43.0)	26020 (89.5)	3050 (10.5)	18480 (63.6)	10590 (36.4)	1817 (6.3)	27253 (93.7)	12258 (42.2)	16812 (57.8)	17462 (60.1)	11608 (39.9)	23413 (80.5)	5657 (19.5)
Albania, 2017-2018	3242 (43.5)	4217 (56.5)	152 (2.02)	7307 (97.97)	6735 (90.3)	724 (9.7)	134 (1.8)	7325 (98.2)	4998 (67.0)	2461 (33.0)	5489 (73.6)	1970 (26.4)	119 (1.6)	7340 (98.4)	4002 (53.7)	3457 (46.3)	5733 (76.9)	1726 (23.1)	4460 (59.8)	2999 (40.2)
Bangladesh, 2014	5665 (34.0)	10981 (66.0)	8539 (51.3)	8107 (48.7)	12983 (78.0)	3663 (22.0)	4528 (27.2)	12118 (72.8)	11603 (69.7)	5043 (30.3)	13161 (79.1)	3485 (20.9)	1957 (11.8)	14689 (88.2)	6124 (36.8)	10522 (63.2)	11418 (68.6)	5228 (31.4)	6262 (37.6)	10384 (62.4)
Cambodia, 2014	2527 (29.9)	5920 (70.1)	1360 (16.1)	7087 (83.9)	6895 (81.6)	1552 (18.4)	902 (10.7)	7545 (89.3)	2251 (26.6)	6196 (73.4)	3555 (42.1)	4892 (57.9)	325 (3.8)	81822 (96.2)	3172 (37.6)	5275 (62.4)	5099 (60.4)	3348 (39.6)	4224 (50.0)	4223 (50.0)
Egypt, 2014	9515 (44.2)	12077 (55.8)	4815 (22.4)	16727 (77.6)	16969 (78.8)	4573 (21.2)	3519 (16.3)	18023 (83.7)	18156 (84.3)	3386 (15.7)	13200 (61.3)	8342 (38.7)	730 (3.4)	20812 (96.6)	7898 (36.7)	13644 (63.3)	14440 (67.0)	7102 (33.0)	9975 (46.3)	11567 (53.7)
Ghana, 2014	1486 (45.9)	1748 (54.1)	1090 (33.7)	2144 (66.3)	2203 (68.1)	1031 (31.9)	886 (27.4)	2348 (72.6)	487 (15.1)	2747 (84.9)	1188 (36.7)	2046 (63.3)	70 (2.2)	3164 (97.8)	1483 (45.9)	1751 (54.1)	1561 (48.3)	1673 (51.7)	2433 (75.2)	801 (24.8)
Haiti, 2016-2017	1732 (34.2)	3330 (65.8)	1130 (22.3)	3932 (77.7)	3964 (78.3)	1098 (21.7)	930 (18.4)	4132 (81.6)	2092 (41.3)	2970 (58.7)	3817 (75.4)	1245 (24.6)	189 (3.7)	4873 (96.3)	2280 (45.0)	2782 (55.0)	3207 (63.4)	1855 (36.6)	3327 (65.7)	1735 (34.3)
India, 2015-2016	3242 (43.5)	4217 (56.5)	52 (.7)	7407 (99.3)	6735 (90.3)	724 (9.7)	134 (1.8)	7325 (98.2)	4998 (67.0)	2461 (33.0)	5489 (73.6)	1970 (26.4)	119 (1.6)	7340 (98.4)	4002 (53.7)	3457 (46.3)	5733 (76.3)	1726 (23.1)	4460 (59.8)	2999 (40.2)
Indonesia, 2017	17221 (50.3)	17027 (49.7)	710 (2.1)	33538 (97.9)	28516 (83.3)	5732 (16.7)	615 (1.8)	33633 (98.2)	14725 (43.0)	19523 (57.0)	14939 (43.6)	19309 (56.4)	666 (1.9)	33582 (98.1)	14594 (42.6)	19654 (57.4)	19409 (56.7)	14839 (43.3)	13472 (39.3)	20776 (60.7)
Jordan, 2017	5350 (79.7)	1365 (20.3)	222 (3.3)	6493 (96.7)	5686 (84.7)	1029 (15.3)	252 (3.8)	6463 (96.2)	5866 (87.4)	849 (12.6)	1618 (24.1)	5097 (75.9)	190 (2.8)	6252 (97.2)	3700 (55.1)	3015 (44.9)	4192 (62.4)	2523 (37.6)	3281 (48.9)	3434 (51.1)
Kenya, 2014	3808 (36.5)	6623 (63.5)	1787 (17.1)	8644 (82.9)	7240 (69.4)	3191 (30.6)	1415 (13.6)	9016 (86.4)	3457 (33.1)	6974 (66.9)	2981 (28.6)	7450 (71.4)	379 (3.6)	10052 (96.4)	4686 (44.9)	5745 (55.1)	5921 (56.8)	4510 (43.2)	5288 (50.7)	5143 (49.3)
Maldives, 2016-2017	996 (12.93)	6703 (87.14)	364 (4.72)	7335 (95.27)	6446 (83.72)	1253 (16.27)	528 (9.27)	5092 (90.6)	4575 (59.42)	3124 (40.57)	4822 (62.63)	2877 (37.36)	1015 (13.18)	6684 (8.67)	4234 (54.99)	3465 (45.00)	3964 (51.48)	3735 (48.51)	6574 (85.64)	1125 (14.61)
Nepal, 2016	3142 (62.32)	1820 (36.67)	2037 (41.05)	2925 (58.94)	3483 (70.19)	1479 (29.80)	757 (15.25)	4205 (84.74)	1937 (39.03)	3025 (60.96)	2471 (49.79)	2491 (50.20)	379 (7.63)	4583 (92.36)	2069 (41.69)	2893 (58.30)	3738 (75.33)	1224 (24.66)	2386 (48.08)	2576 (51.91)
Pakistan, 2017-2018	2478 (48.2)	2662 (51.79)	2599 (50.56)	2541 (49.43)	3706 (72.10)	1434 (27.89)	1404 (27.31)	3736 (72.68)	4503 (87.60)	637 (12.39)	4033 (78.46)	1107 (21.53)	253 (4.92)	4887 (95.07)	2107 (40.99)	3033 (59.00)	2885 (56.12)	2255 (43.87)	3419 (6651)	1721 (33.48)
Philippines, 2017	9016 (35.95)	16058 (64.04)	313 (1.24)	24761 (98.75)	21201 (84.55)	3873 (15.44)	279 (1.80)	15166 (98.19)	13699 (54.63)	11375 (45.36)	8721 (34.78)	16353 (65.21)	5120 (20.41)	19954 (79.58)	11422 (45.55)	13652 (54.44)	14255 (56.85)	10819 (43.14)	16642 (66.37)	8432 (33.62)
Senegal, 2017	7506 (44.71)	9279 (55.28)	8470 (50.46)	8315 (49.53)	2878 (17.14)	13907 (82.85)	7392 (44.03)	4001 (23.83)	8613 (51.32)	8172 (48.68)	7287 (43.41)	9498 (56.58)	3919 (23.34)	12866 (76.65)	7424 (44.22)	9361 (55.77)	2878 (17.14)	13907 (82.85)	13728 (81.78)	3057 (18.21)
Tanzania, 2015-2016	4142 (31.24)	9116 (68.75)	1998 (15.07)	11260 (84.92)	9678 (72.99)	3580 (27.00)	1062 (12.97)	7124 (87.02)	3771 (28.44)	9487 (71.55)	4722 (35.61)	8536 (64.38)	2927 (22.07)	10331 (77.92)	3671 (27.68)	9587 (72.31)	3996 (30.14)	9262 (69.85)	9403 (70.92)	3855 (29.07)
Timor-Leste, 2016	4337 (34.40)	8269 (65.59)	2592 (20.56)	9914 (78.64)	10296 (81.67)	2310 (18.32)	1997 (26.41)	5562 (73.58)	8104 (64.57)	4502 (35.71)	9370 (74.32)	3236 (25.67)	3126 (24.79)	9480 (75.20)	4378 (37.72)	8228 (65.27)	8905 (70.64)	3701 (29.35)	10630 (84.32)	1976 (15.67)

**Table 2:** Binary logistic regression model estimation for Bangladesh for different influencing factors of contraceptive use.

Variable	OR	*p* value	S. E.	95% CI for OR	
				Lower	Upper
*Type of place of residence*					
Urban (ref)					
Rural	0.73	0.000	0.039	0.68	0.79
*Respondent education level*					
No education (ref)					
Primary	1.25	0.000	0.049	1.14	1.38
Secondary	1.45	0.000	0.054	1.30	1.61
Higher	1.63	0.000	0.084	1.38	1.92
*Currently breastfeeding*					
No (ref)					
Yes	1.70	0.000	0.043	1.56	1.85
*Husband education level*					
No education (ref)					
Primary	1.06	0.217	0.047	0.97	1.16
Secondary	0.98	0.302	0.052	0.86	1.05
Higher	1.01	0.890	0.071	0.88	1.16
*Respondent currently working*					
No (ref)					
Yes	1.47	0.000	0.037	1.37	1.58
*Media exposure*					
No (ref)					
Yes	1.08	0.075	0.043	0.99	1.18
*Respondent age*					
15-19 (ref)					
20-25	1.18	0.004	0.059	1.05	1.33
26-49	1.27	0.000	0.064	1.12	1.44
*Wealth index*					
Rich (ref)					
Middle	0.87	0.002	0.047	0.79	0.95
Poor	0.75	0.000	0.047	0.68	0.82
*Desire for more children*					
No (ref)					
Yes	0.50	0.000	0.044	0.46	0.55

**Table 3:** Random effects model estimation of OR for 18 developing countries.

Country names	Type of place of residence	Respondent education	Breastfeeding	Husband education	Respondent currently working	Media exposure	Respondent age	Wealth index	Desire for more children
	OR	OR	OR	OR	OR	OR	OR	OR	OR
Afghanistan, 2015	0.52	1.47	1.15	1.36	0.87	2.00	3.63	1.76	0.65
Albania, 2017-2018	0.96	2.03	0.83	2.98	1.81	1.03	1.33	1.10	0.74
Bangladesh, 2014	0.80	1.00	1.42	0.96	1.50	1.08	1.66	0.91	0.52
Cambodia, 2014	1.01	1.31	1.07	1.28	1.49	1.07	3.24	1.15	0.78
Egypt, 2014	0.82	1.15	1.81	1.23	1.24	1.16	4.90	1.35	0.33
Ghana, 2014	1.09	1.76	0.93	1.88	1.25	—	1.21	1.25	0.95
Haiti, 2016-2017	0.83	1.34	0.91	1.19	1.22	1.29	1.69	1.19	0.73
India, 2015-2016	0.96	2.03	1.81	2.98	1.81	1.08	1.33	1.10	0.74
Indonesia, 2017	1.00	2.56	1.94	1.78	1.02	1.14	1.99	1.09	0.40
Jordan, 2017	1.09	1.98	1.42	2.60	1.10	1.48	6.05	1.28	0.40
Kenya, 2014	0.73	—	0.91	—	2.25	4.50	2.51	—	0.68
Maldives, 2016-2017	1.18	0.63	1.73	0.78	1.48	1.29	3.96	0.90	0.38
Nepal, 2016	0.83	0.92	0.58	0.95	1.54	1.42	3.86	1.08	0.20
Pakistan, 2017-2018	0.64	1.84	0.97	1.61	1.24	1.67	4.63	2.14	0.25
Philippines, 2017	1.17	2.02	2.88	2.41	1.59	1.46	13.07	0.98	0.33
Senegal, 2017	0.68	1.03	2.07	1.81	1.59	2.16	9.11	1.33	0.38
Tanzania, 2015-2016	0.67	1.50	1.31	2.70	2.23	1.72	5.76	1.51	0.59
Timor-Leste, 2016	1.15	1.08	2.35	1.52	1.81	1.67	6.06	1.06	1.80
*I* ^2^ (%)	97.2%	95.2%	98.6%	95.5.%	97.4%	98.6%	98.4%	96.6%	99.1%
*τ* ^2^	0.05	0.11	0.16	0.15	0.06	0.13	0.61	0.05	0.18

**Table 4:** Random effects model estimation (summary effect) for different variables on 18 developing countries.

Variables	Random effects model			
	Odds ratio (OR)	*p* value	Confidence interval	
			Lower bound	Upper bound
Type of place of residence	0.88	0.0213	0.78	0.98
Respondent education level	1.39	0.0001	1.17	1.65
Currently breastfeeding	1.34	0.0022	1.11	1.62
Husband education level	1.60	0.0001	1.32	1.93
Respondent currently working	1.47	0.0001	1.30	1.66
Media exposure	1.48	0.0001	1.25	1.76
Respondent age	3.41	0.0001	2.35	4.93
Wealth index	1.21	0.0004	1.10	1.35
Desire for more children	0.53	0.0001	0.43	0.65

## Data Availability

The underlying data (https://dhsprogram.com/data/available-datasets.cfm) are available under the terms of the Creative Commons Attribution 4.0 International license (CC-BY 4.0).

## Supplementary Materials

Supplementary Materials The appendix (1-8) in the supplementary file shows the overall effect estimate changes after removing one study. The overall effect size was calculated, omitting one country at a time.
