# Supplementary material for: Exploring the Influencing Factors for Contraceptive Use among Women: A Meta-Analysis of Demographic and Health Survey Data from 18 Developing Countries
Source: Int J Reprod Med. 2022 Nov 14;2022:6942438. doi: 10.1155/2022/6942438 (PMC9678470; doi:10.1155/2022/6942438)
Supplement: Supplementary Materials — The appendix (1-8) in the supplementary file shows the overall effect estimate changes after removing one study. The overall effect size was calculated, omitting one country at a time. [file 6942438.f1.doc]

The appendix (1-8) shows the overall effect estimate changes after removing one study. The overall effect size was calculated, omitting one country at a time. The leave-one-out analysis reported evidence that husband’s education, respondent’s education and wealth index of Kenia 2014, had greatest influence on pooled estimated of contraceptive use from Appendix 2, 4, 6. Appendix 3 exhibited that the pooled effect size is influenced by the Media Access of Ghana 2014. Omitting each country does not influence the overall effect size, presented in Appendix 1, 5, 7, 8, and 9.

**Appendix 1 Results of Sensitivity Analysis for Age**


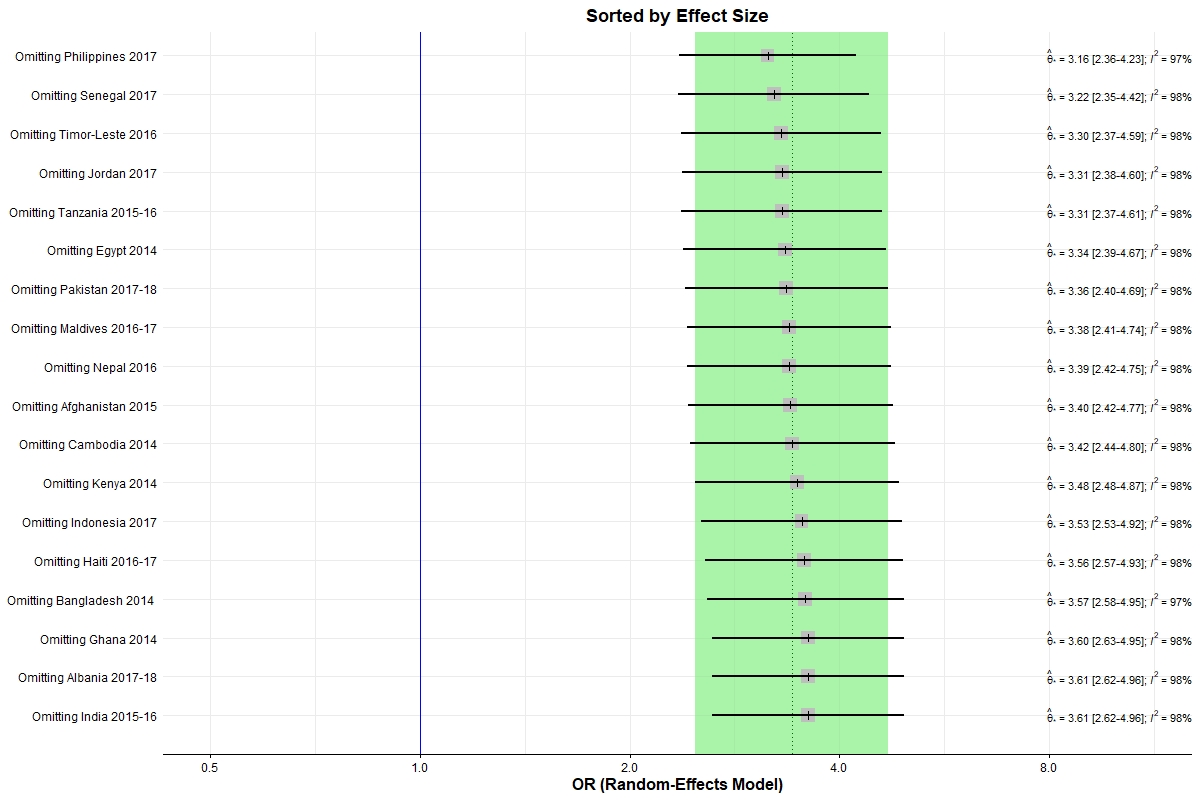


**Influence of each country on the overall pooled estimate from leave-one-country-out sensitivity analysis**

**Appendix 2 Results of Sensitivity Analysis for Husband Education**


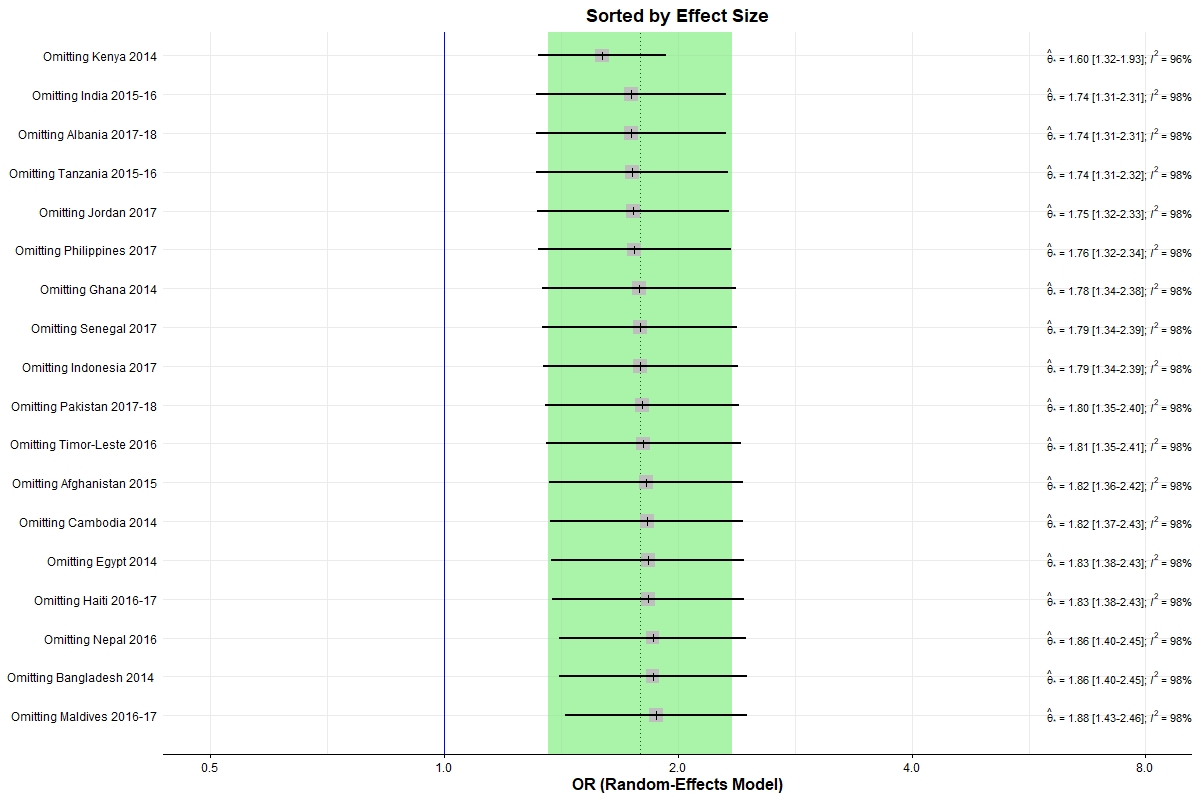


**Influence of each country on the overall pooled estimate from leave-one-country-out sensitivity analysis**

**Appendix 3 Results of Sensitivity Analysis for Media Access**


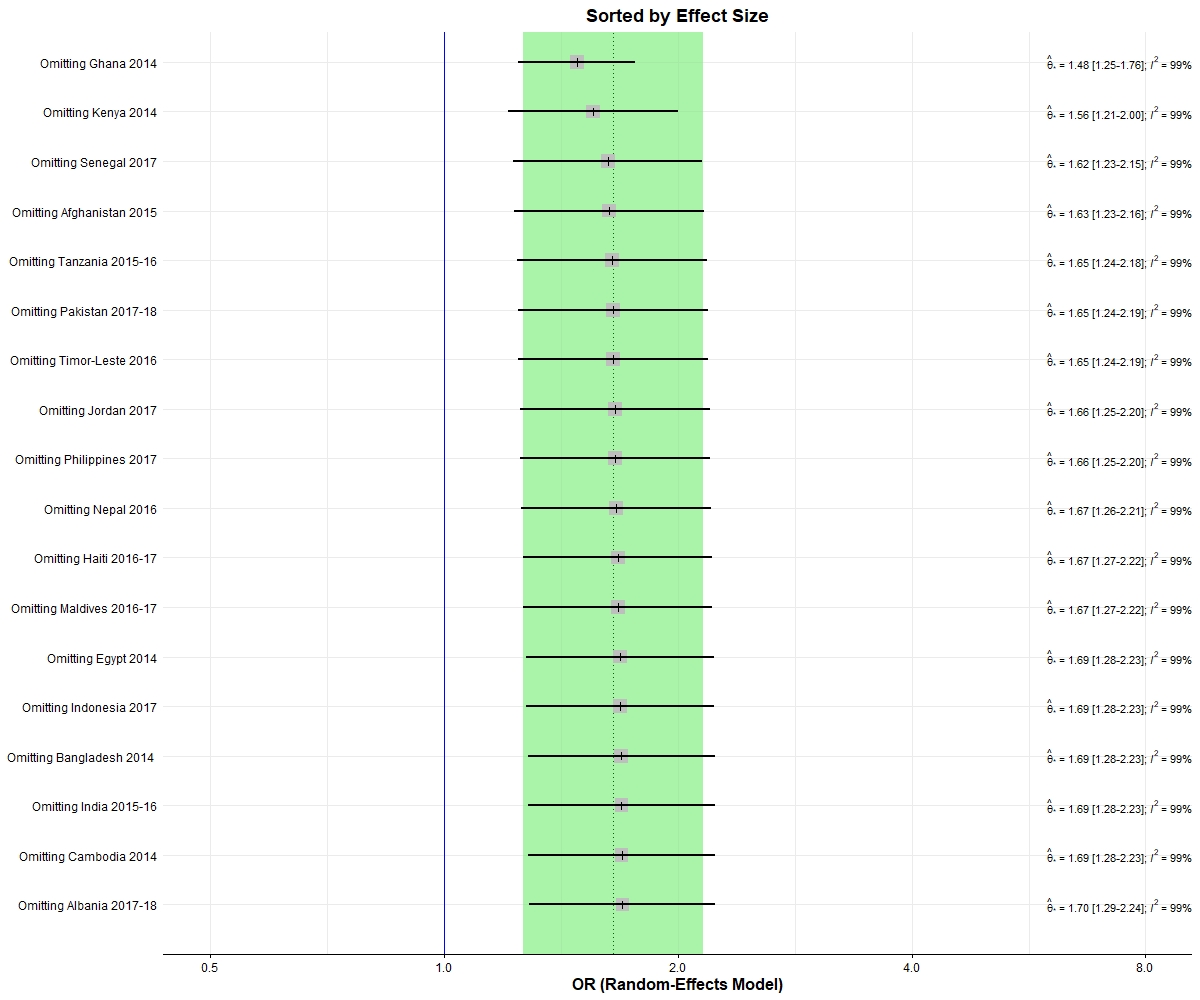


**Influence of each country on the overall pooled estimate from leave-one-country-out sensitivity analysis**

**Appendix 4 Results of Sensitivity Analysis for Respondent Education**


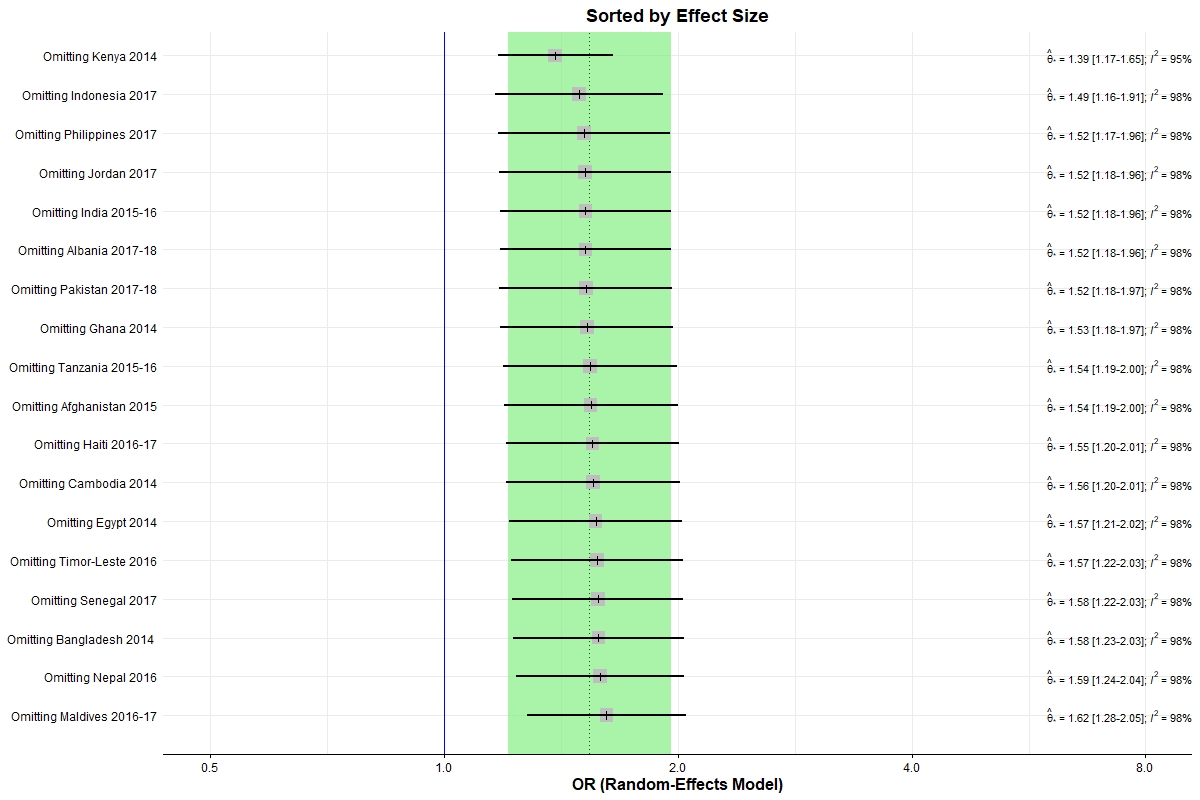


**Influence of each country on the overall pooled estimate from leave-one-country-out sensitivity analysis**

**Appendix 5 Results of Sensitivity Analysis for Type of Place of Residence**


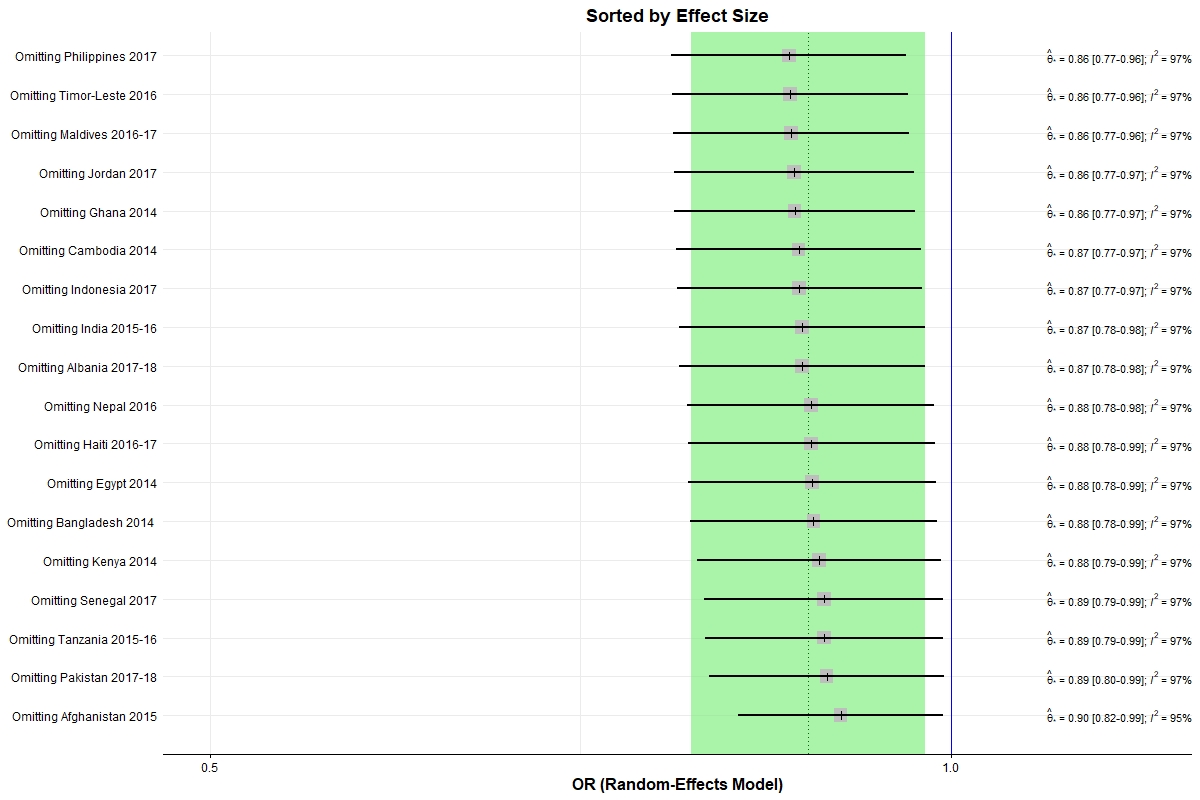


**Influence of each country on the overall pooled estimate from leave-one-country-out sensitivity analysis**

**Appendix 6 Results of Sensitivity Analysis for Wealth Index**


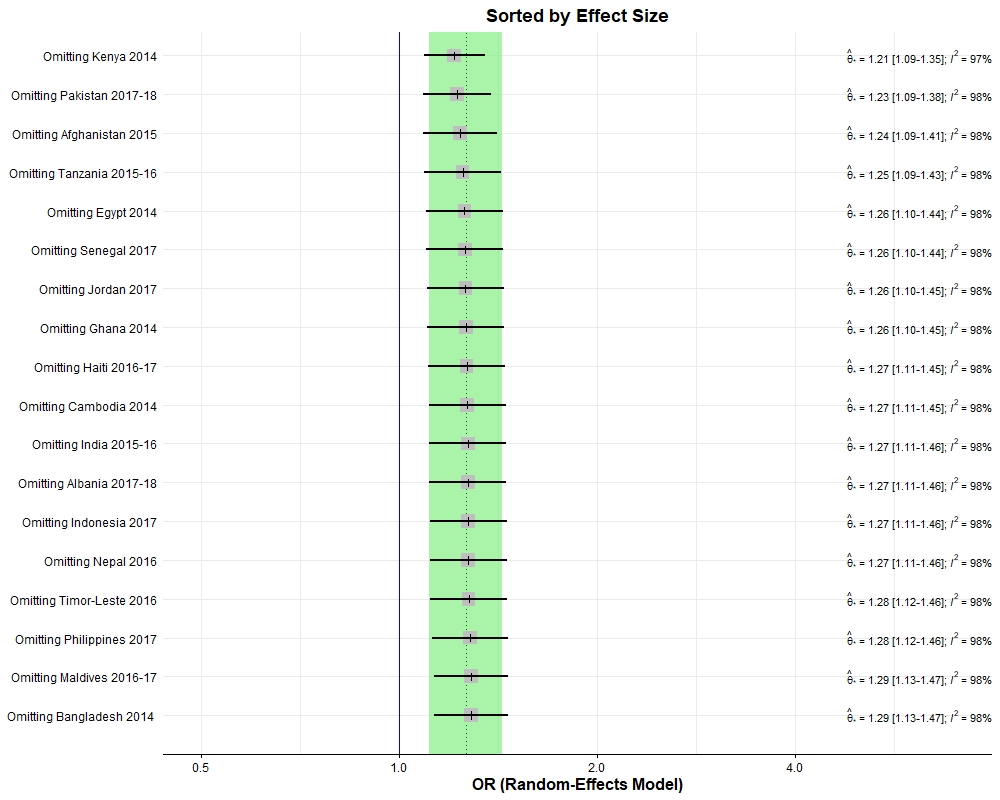


**Influence of each country on the overall pooled estimate from leave-one-country-out sensitivity analysis**

**Appendix 7 Results of Sensitivity Analysis for Working Status**


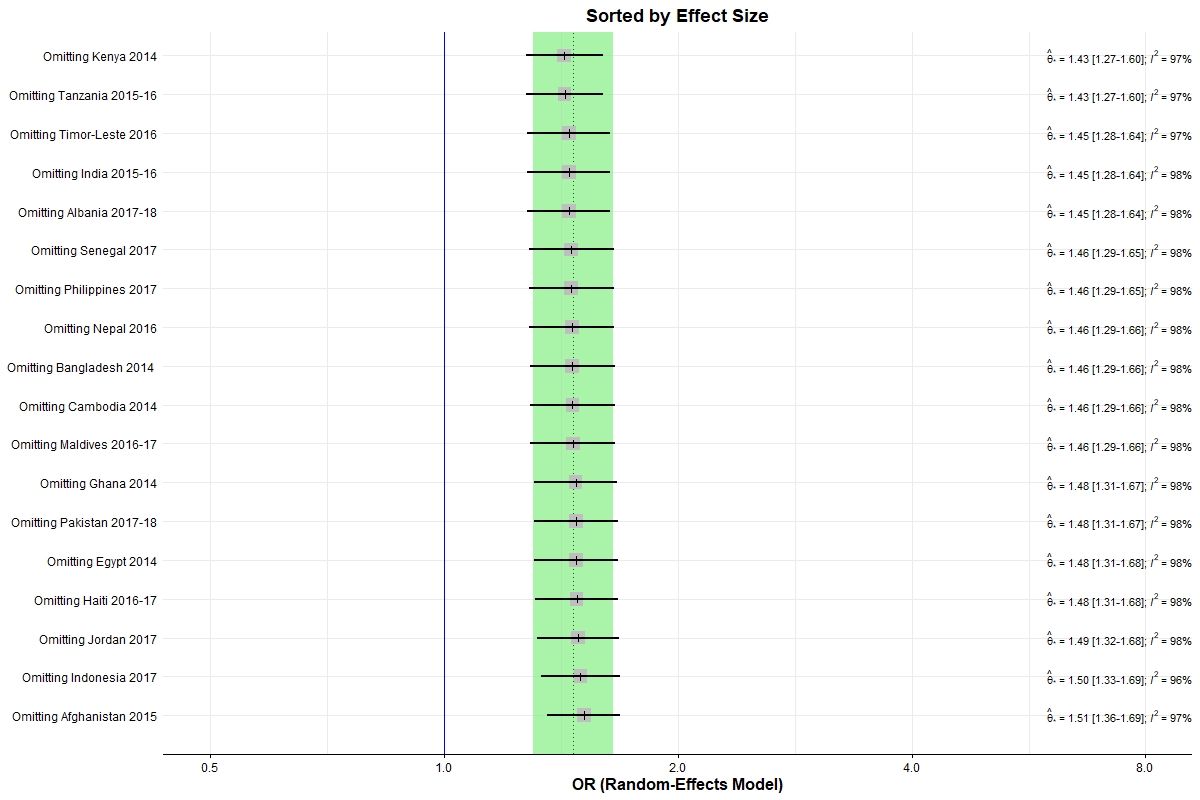


**Influence of each country on the overall pooled estimate from leave-one-country-out sensitivity analysis**

**Appendix 7 Results of Sensitivity Analysis for Desire for More Children**


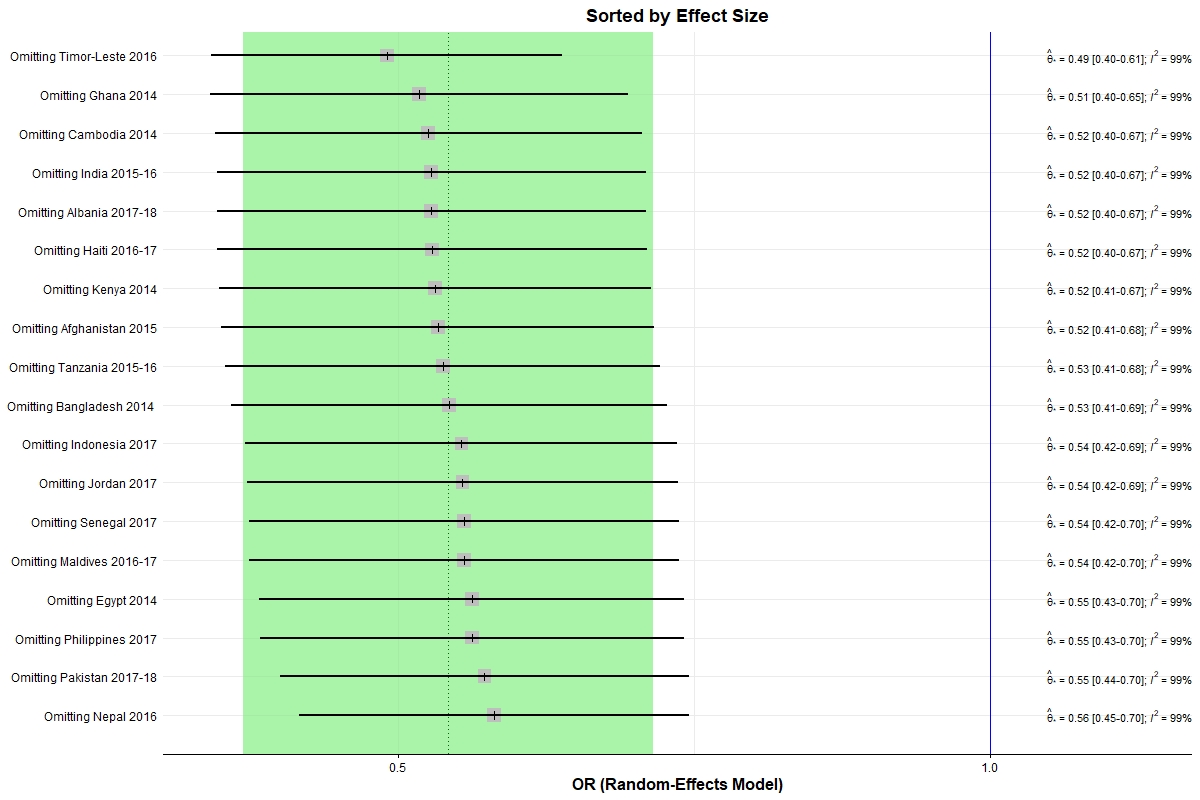


**Influence of each country on the overall pooled estimate from leave-one-country-out sensitivity analysis**

**Appendix 8 Results of Sensitivity Analysis for Breastfeeding Status**


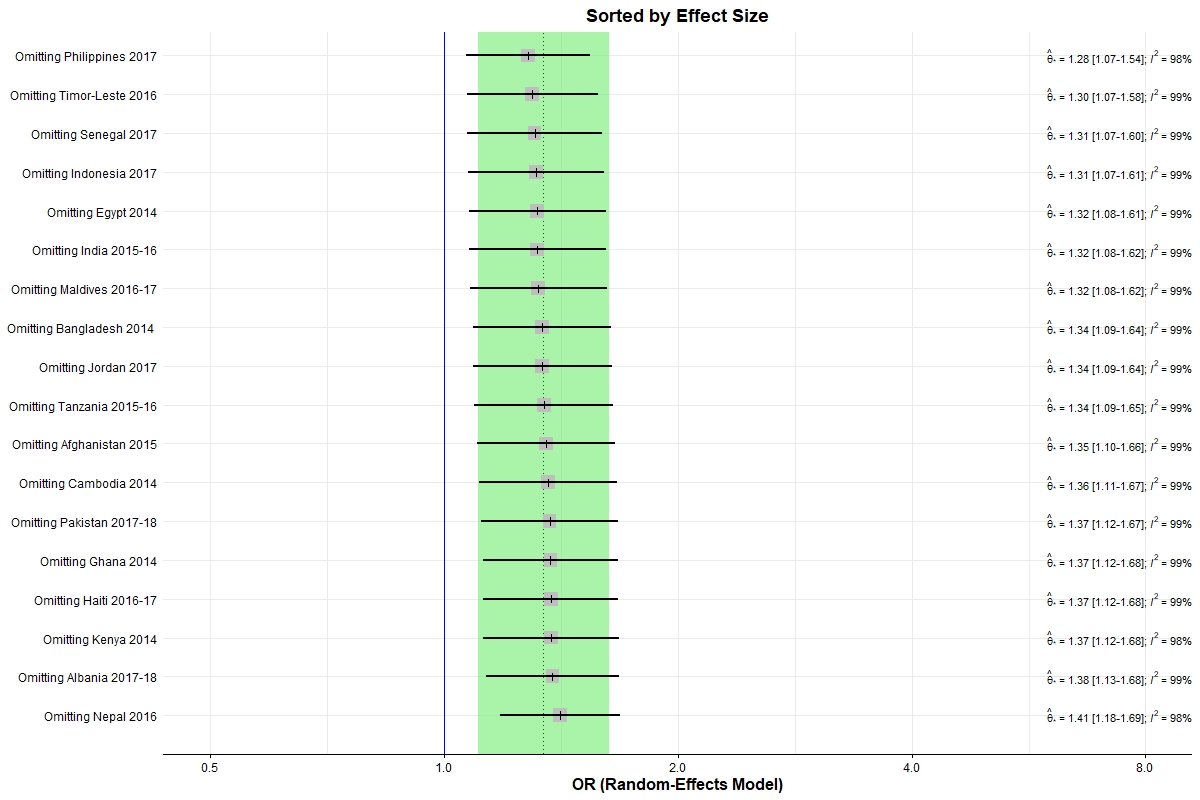


**Influence of each country on the overall pooled estimate from leave-one-country-out sensitivity analysis**
